# miR-23a-3p is a Key Regulator of IL-17C-Induced Tumor Angiogenesis in Colorectal Cancer

**DOI:** 10.3390/cells9061363

**Published:** 2020-06-01

**Authors:** Yunna Lee, Su Jin Kim, Jieun Choo, Gwangbeom Heo, Jin-Wook Yoo, Yunjin Jung, Sang Hoon Rhee, Eunok Im

**Affiliations:** 1 College of Pharmacy, Pusan National University, Busan 46241, Korea; yunnalee@pusan.ac.kr (Y.L.); sjsyk@pusan.ac.kr (S.J.K.); jieunchoo@pusan.ac.kr (J.C.); gbheo@pusan.ac.kr (G.H.); jinwook@pusan.ac.kr (J.-W.Y.); jungy@pusan.ac.kr (Y.J.); 2 Department of Biological Sciences, Oakland University, Rochester, MI 48309, USA; srhee@oakland.edu

**Keywords:** microRNA, interleukin-17C, angiogenesis, vascular endothelial growth factor, colorectal cancer

## Abstract

MicroRNAs (miRNAs) have emerged as key players in tumor angiogenesis. Interleukin-17C (IL-17C) was identified to promote colorectal cancer (CRC) progression. Therefore, we aimed to investigate the effect of IL-17C on tumor angiogenesis, the involvement of miR-23a-3p in IL-17C signaling, and the direct target gene of miR-23a-3p in CRC. In vitro and ex vivo angiogenesis, a mouse xenograft experiment, and immunostaining were performed to test the effect of IL-17C on tumor angiogenesis. ELISA, quantitative real time PCR, and gene silencing were used to uncover the underlying mechanism. IL-17C induced angiogenesis of intestinal endothelial cells, subsequently enhancing cell invasion and migration of DLD-1 cells. IL-17C-stimulated DLD-1 cells produced vascular endothelial growth factor (VEGF) to enhance angiogenesis. Moreover, IL-17C markedly accelerated xenograft tumor growth, which was manifested by substantially reduced tumor growth when treated with the VEGF receptor 2 inhibitor Ki8751. Accordingly, Ki8751 suppressed the expression of IL-17C-stimulated PECAM and VE-cadherin in xenografts. Furthermore, IL-17C activated STAT3 to increase the expression of miR-23a-3p that suppressed semaphorin 6D (SEMA6D) expression, thereby permitting VEGF production. Taken together, our study demonstrates that IL-17C promotes tumor angiogenesis through VEGF production via a STAT3/miR-23a-3p/SEMA6D axis, suggesting its potential as a novel target for anti-CRC therapy.

## 1. Introduction

Colorectal cancer (CRC) is the third most common cancer type and the major leading cause of cancer death worldwide [1]. Incidence rates are increasing with economic development and westernization of lifestyle in Asian and African countries [2]. The Republic of Korea has the highest age-standardized CRC incidence rate globally [3]. Angiogenesis is particularly vital to tumor growth, as cancer cells require blood vessel growth, which provides abundant oxygen and essential nutrients necessary for cancer cell proliferation [4]. However, the molecular mechanism underlying tumor angiogenesis is still limited.

The interleukin (IL)-17 family is a Th17 cell-derived pro-inflammatory cytokine family involved in numerous human diseases [5]. The IL-17 family consists of six cytokines including IL-17A, IL-17B, IL-17C, IL-17D, IL-17E, and IL-17F [5]. IL-17C is a new relative of IL-17A and its signaling is activated by a receptor complex of IL-17 receptor A (IL-17RA) and IL-17RE [6,7]. Recent clinical and non-clinical studies suggest an association between the IL-17 family and the progression of various tumor types such as gastric, hepatocellular, breast, and colorectal cancer (CRC) [8,9,10,11,12]. The levels of IL-17A and IL-17RA were significantly higher in human CRC than normal colon tissues, which was positively correlated with poor prognosis of CRC [8,13]. Additionally, the angiogenic activity of IL-17A contributes to its tumorigenic activity by increasing the secretion of angiogenic chemokines such as vascular endothelial growth factor (VEGF), CXCL6, and CXCL8 [14]. Noteworthy, dysregulated microbiota and microbiota-mediated toll-like receptor (TLR)/MyD88-dependent signaling in pathogenic states upregulate IL-17C expression in human and mouse CRC tissues by increasing intestinal epithelial cells (IEC) survival [12]. Therefore, exploring the role of IL-17C in CRC progression and its possible function in tumor angiogenesis expands our understanding of CRC development.

MicroRNAs (miRNAs) have recently gained popularity as important posttranscriptional modulators of various biological processes and pathophysiology of human diseases [15,16]. Some miRNAs have emerged as key players in angiogenesis. These angiogenesis-related miRNAs are abundant in the vascular system and are involved in regulation of endothelial function and angiogenesis [17,18,19]. The representative miRNAs include miR-126, miR-221/222, the miR-23~27~24 cluster, and the miR-17~92 cluster [20]. Among them, the miR-23~27~24 cluster is highly expressed in endothelial cells and called “AngiomiRs” [17]. The knockdown of the miR-23~27~24 cluster using locked nucleic acid (LNA)-anti-miR impaired angiogenesis in vitro and retinal vascular development in vivo [21]. The silencing miR-23~27~24 repressed endothelial cell proliferation, despite VEGF stimulation by impairment of the mitogen-activated protein kinases (MAPKs) and phosphoinositide-3-kinase (PI3K)-AKT signaling pathways. The pro-angiogenic functions of the miR-23~27~24 cluster are closely linked to genetic repression of the target genes, spouty2 and semaphorin (SEMA) 6A, which exert antiangiogenic activity [21]. Therefore, the precise role of angiogenesis-related miRNA in tumor angiogenesis remains to be elucidated.

Alteration in gut microbiota can trigger IL-17C upregulation, which promotes tumor progression by increasing IEC survival in an autocrine manner [12]. Moreover, IL-17A can modulate angiogenesis in multiple cancer types. However, the angiogenic effect of IL-17C on CRC remains largely unclear.

This study aimed to determine biological function and mechanistic action of IL-17C involving miRNA in CRC progression and angiogenesis. Accordingly, the primary objectives of this study include 1) to determine whether IL-17C plays a role in tumor angiogenesis and progression of CRC; 2) to uncover whether miRNAs are involved in IL-17C signaling, thereby regulate pro-angiogenic VEGF production. Angiogenic and tumorigenic activity of IL-17C was measured by using in vitro tube formation assays and ex vivo aortic ring assays in intestinal endothelial cells and a mouse xenograft model with DLD-1 cells. The underlying mechanism by which miRNAs are involved in IL-17C-induced VEGF production was determined by using gene silencing technique.

## 2. Materials and Methods

### 2.1. Cell Culture and Transfection

Human intestinal microvascular endothelial cells (HIMECs) were isolated as previously described [22]. HIMECs were cultured in endothelial cell growth media kits (EGM-2 BulletKit) (Lonza, Walkersville, MD, USA) supplemented with 2% fetal bovine serum (FBS, HyClone Laboratories, Logan, UT, USA) and 100 unit/mL penicillin-streptomycin solution (HyClone Laboratories) on fibronectin (4 μg/cm^2^; Sigma-Aldrich, St Louis, MO, USA) coated plates. HIMECs were maintained at 37 °C in 5% CO_2_. For all experiments, cells were used after three to approximately 10 passages. Human non-transformed human colonic epithelial (NCM460) cells were obtained from INCELL Corp (San Antonio, TX, USA) and human colon carcinoma (Caco-2, HT-29, DLD-1, and HCT116) cells were purchased from the American Type Culture Collection (Manassas, VA, USA). Cells were cultured in DMEM with high glucose or RPMI1640 supplemented with 10% heat-inactivated FBS and 100 unit/mL penicillin-streptomycin solution at 37 °C in a 5% CO_2_ atmosphere. Cells were transfected at 70–80% confluence in a six-well plate using Lipofectamine RNAiMax (Invitrogen Life Technologies, Gaithersburg, MD, USA), following the manufacturer´s instructions. Briefly, 25 pmole/well Lipofectamine RNAiMAX reagent was diluted in Opti-MEM medium (Gibco BRL, Gaithersburg, MD, USA) and 30 pmole/well siRNA was diluted in Opti-MEM medium. Next, the diluted siRNA and diluted Lipofectamine RNAiMAX reagent were mixed to 1:1 ratio and incubate for 5 min. The mixture of siRNA-lipid complex was added to cells. For miR-23a-3p inhibition, 50 nmol/L of miR-23a-3p inhibitors or their negative controls were used (Bioneer, Daejeon, South Korea). Signal transducer and activator of transcription 3 (STAT3) inhibition was achieved by transfection using 30 pmol/L of STAT3 siRNA and SEMA6D inhibition was achieved by transfection using 30 pmol/L of SEMA6D siRNA or negative control siRNA (Bioneer). Forty-eight hours after transfection, the cells were trypsinized, harvested, and used for the experiments.

### 2.2. RNA Preparation and Microarray

HIMECs were seeded into 100 mm cell culture plates (2 × 10^6^ cells/well) and stabilized for 24–36 h. HIMECs were stimulated with 100 ng/mL human IL-17C (eBioscience, San Diego, CA, USA) for 3 h, and harvested after incubation. Total RNA was isolated using the RNeasy plus mini kit (Qiagen, MD, USA). RNA samples (1000 ng) were reversely transcribed to complementary DNA (cDNA) using the RT & GO mastermix (MPbio, Santa Ana, CA, USA) and oligo (dT) primers (ELPIS-Biotech, Daejeon, South Korea). Microarray was performed in the RT^2^ Profiler PCR Array plate PAHS-169ZA (Qiagen), for screening the genes involved in human Crohn’s disease. Microarray and quantitative real-time PCR (qPCR) were performed using the 96-well plate format in Thermal cycler TP800 (TAKARA, Otsu, Japan).

### 2.3. Reverse Transcription PCR (RT-PCR) and qPCR

To detect IL-17C expression in HIMECs, NCM460, and DLD-1 cells, RT-PCR was conducted. For the IL-17RA and IL-17RE detection, HIMECs were stimulated by 100 ng/mL human IL-17C for 3 h. The expression of IL-17RA and IL-17RE receptors was detected in NCM460 and DLD-1 cells without any stimulation. The expression IL-17C and its receptors were confirmed by using manually designed primer sequences as follows: IL-17C (forward: 5′- CCC TCA GCT ACG ACC CAG T - 3′, reverse: 5′- CTT CTG TGG ATA GCG GTC CT - 3′); IL-17RA (forward: 5′- GCG AAA TAG CGT CCT CTT CC - 3′, reverse: 5′- AGC GAG AGC ATC AAG CCT TC - 3′); IL-17RE (forward: 5′- TCC AGT TTG CCT GGA AGC AC - 3′, reverse: 5′- ACA CCC AGT AGG GTG AG - 3′) and GAPDH (forward: 5′- CGC TCT CTG CTC CTC CTG TT- 3′, reverse: 5′- CCA TGG TGT CTG AGC GAT GT - 3′).

SEMA6D expression was confirmed in miR-23a-3p inhibitor transfected DLD-1 cells by using following sequences, forward: 5′- TTC TGC CAC AGT GGC TGA TT- 3′, reverse: 5′- GCC TTG GTT TTG GTA CTT TG - 3′. Same human GAPDH primer is used for control. The first cDNA strand was amplified using the Maxime PCR PreMix (iNtRON Biotechnology, Gyeonggi-do, South Korea) with initial denaturation for hot-start (94 °C, 10 min), 24 cycles of denaturation (94 °C, 1 min), primer annealing (53 °C, 1 min), extension (72 °C, 45 s), and final extension (72 °C, 10 min). To confirm the microarray, HIMECs were stimulated with 100 ng/mL human IL-17C for 3 h, and qPCR was performed in the Thermal cycler TP800 (TAKARA). Primers used for the qPCR were designed manually and the sequences were as follows: MMP-1 (forward: 5′- GGA CCT GGA AAT CTT GC- 3′, reverse: 5′- GCC ATT CTC TTG GAC TCT CCC - 3′); MMP-10 (forward: 5′- AAG ATC CCA CTG GAA CCC TG - 3′, reverse: 5′- TGG CCC AGA ACT CAT TTC CT - 3′); IL-8 (forward: 5′- ATG ACT TCC AAG CTG GCC GTG GCT- 3′, reverse: 5′- TCT CAG CCC TCT TCA AAA ACT TCT C - 3′); CXCL3 (forward: 5′- TCA CTG AAC TGC GCT GCC AG - 3′, reverse: 5′- TCT TCC CAT TCT TGA GTG TGG C - 3′); CX3CL1 (forward: 5′- TAT CAC TCC TGT CCC TGA CG - 3′, reverse: 5′- GCT CTG GTA GGT GAA CAT GG - 3′); GAPDH used for qPCR were shown above as the same primer for IL-17C RT-PCR. After amplification, the results were analyzed using the Thermal cycler dice real time system v5 (TAKARA). The delta Ct method was performed to analyze mRNA expression.

### 2.4. miRNA Analysis

DLD-1 cells (1 × 10^6^ cells/well) were seeded into 100 mm cell culture plates and stabilized for 24–36 h. IL-17C (100 ng/mL) was treated to the cultured cells for 3 h, and total mRNA was isolated using miRCURY miRNA isolation kits (Exiqon, Woburn, MA, USA). To quantify miRNA transcript levels, qPCR was performed using the miRCURY LNA™ Universal RT microRNA PCR Starter Kit (Exiqon) and miScript Primer Assay (Qiagen), following the manufacturer’s instructions. The primer sequences used to detect miR-23a-3p and the endogenous control miR-103a-3p were as follows: hsa-miR-23a-3p (5′- AUC ACA UUG CCA GGG AUU UCC -3′); hsa-miR-103a-3p (5′- AGC AUU GUA CAG GGC UAU GA -3′).

### 2.5. 3-(4,5-Dimethylthiazol-2-yl)-2,5-diphenyltetrazolium bromide (MTT) Assay

DLD-1 cells were seeded into 96-well plates at a density of 1 × 10^3^ cells/well and stabilized for 24 h. DLD-1 cells were then treated with IL-17C (100 ng/mL; eBioscience) for 24 h. Subsequently, 20 µL of MTT solution (5 mg/mL) (Duchefa Biochemie, Haarlem, The Netherlands) was added to each well and incubated for 4 h. The MTT solution was then removed, and 150 µL of DMSO was added to each well to dissolve the formazan crystals. Absorbance was measured at 540 nm using the Multiskan™ GO Microplate Spectrophotometer (Thermo Fisher Scientific, Fair Lawn, NJ, USA).

### 2.6. Clonogenic Assay

For the clonogenic assay, DLD-1 cells were seeded at a density of 2 × 10^3^ cells/well into a 24-well plate. Ten days later, colonies were stained with 1% crystal violet for 30 min. Samples were then washed with distilled water and air-dried. Stained cells were counted using the ImageJ v.1.47 software (https://imagej.nih.gov/ij).

### 2.7. Invasion Assay

The invasion assay was performed using Cultrex 24-well basement membrane extract (BME) Cell Invasion Assay kits (Trevigen Inc., Gaithersburg, MD, USA), following the manufacturer’s instructions. Briefly, starved DLD-1 cells are seeded in BME-coated invasion chambers in 10% FBS-DMEM, (bottom chamber) with/without 100 ng/mL IL-17C for 48 h. After incubation, the bottom chambers were dissociated with Cell Dissociation Solution/Calcein-AM. The fluorescence intensity of calcein was detected to quantitate the number of invading cells, using a fluorescence plate reader (GloMax, Promega, WI, USA) at 485 nm excitation and 530 nm emission.

### 2.8. Wound Healing Assay

HIMECs were seeded at a density of 1 × 10^5^ cells/well into a fibronectin-coated 24-well plate and stabilized for 48 h. A scratched wound was created with a plastic pipette tip and washed with phosphate-buffered saline (PBS) for cell debris removal. The cells were then incubated with medium containing 100 ng/mL human IL-17C (eBioscience) for 24 h. DLD-1 cells were seeded at a density of 1 × 10^6^ cells into a 24-well plate, stabilized for 48 h, and supplemented with IL-17C for 24 h. Conditioned medium (CM) was obtained from NCM460 and DLD-1 cells incubated with/without IL-17C for 8 h. HIMECs were wounded and supplemented with each CM for 12 h. Cells were pretreated with Ki8751 (4.7 g/mL; Calbiochem, Gibbstown, NJ, USA) for 30 min, and then, CM with/without IL-17C were added for additional 8 h. HIMECs were wounded and supplemented with each CM for 20 h. Cell migration and wound closure were calculated as the percentage of the total wound area at 24 h to the initial wound area. Three different areas from each group were photographed using the Nikon ECLIPSE TE 2000-U microscope (Nikon, Tokyo, Japan). The wound area was analyzed using ImageJ v1.47 software.

### 2.9. Tube Formation Assay

For this assay, 400 μL of matrigel (BD Biosciences, San Jose, CA, USA) was added to each well of the 24-well plates and incubated at 37 °C for 3 h. After polymerization, 1 × 10^5^ HIMECs were seeded into each well and incubated with endothelial cell growth media (Lonza) with/without 100 ng/mL human IL-17C (eBioscience). After 4 h, tube formation was observed and photographed using the Nikon ECLIPSE TE 2000-U microscope (Nikon). To explore the effect of DLD-1 CM on angiogenesis, HIMECs were plated on Matrigel, supplemented with normal and IL-17C-induced CM from DLD-1 cells for 6 h, and photographed by a Nikon ECLIPSE TE 2000-U microscope (Nikon). The total tube length in each well was measured using ImageJ v.1.47 software.

### 2.10. Aortic Ring Assay

This assay was performed using the Matrigel sandwich method. To polymerize the lower gel layer, 400 μL of Matrigel (BD biosciences) was placed into each well of the 24-well plates and incubated at 37 °C for 3 h. The thoracic aortas of 3-month-old male C57BL6/N mice (*n* = 3) were dissected out. After removing the surrounding fat tissues and rinsing with cold sterile PBS in petri dishes, the aortas were cut into 1 mm ring segments and placed in a lower layer of Matrigel. For the upper gel layer, 100 μL of Matrigel was added on top of each ring using pre-cooled pipette tips and incubated at 37 °C for 1 h. After solidification, the aortic rings were supplemented with endothelial cell growth media (Lonza) with/without 100 ng/mL mouse IL-17C (eBioscience). The aortic rings were grown on Matrigel for 14 days (d), and culture medium was replaced every other day. Aortic vessel outgrowth was monitored daily and photographed using the Nikon ECLIPSE TE 2000-U microscope (Nikon). The average vessel length was calculated using ImageJ v.1.47 software.

### 2.11. Rhodamine-Phalloidin Staining

HIMECs were seeded at a density of 1 × 10^3^ cells/well on fibronectin-coated glass chamber slides (LabTek, Thermo Fisher Scientific). The cells were stabilized at 37 °C for 40 h, and treated with 100 ng/mL human IL-17C (eBioscience) for 15 min. The staining was performed as previously described [22]. Briefly, the cells were then washed with PBS, fixed with 10% formalin (Sigma-Aldrich) for 15 min, and permeabilized with Triton X-100 (0.1%, Daejung, Gyeonggi-do, South Korea) for 5 min. After permeabilization, the cells were washed twice and stained for 15 min in the dark with rhodamine–phalloidin (100 nM/well, Cytoskeleton, Denver, CO, USA). The cells were then washed three times, mounted on slides with 50% glycerol, and examined with fluorescence microscopy Axioskop FL (Carl Zeiss Meditec, Inc., Dublin, CA, USA), using Metamorph Microscopy Automation and Image Analysis Software (Molecular devices, Sunnyvale, CA, USA).

### 2.12. In Vivo Xenograft Mouse Model

A total of 40 female Balb/c nude mice (four weeks of age) were obtained from Orient Bio (Seognam, South Korea) and randomly divided into four groups. The mice were housed under a 12 h light/dark cycle and fed rodent chow (Samtako Bio Korea, Osan, South Korea) and tap water ad libitum. After a 1-week acclimation period, the mice were subcutaneously inoculated with DLD-1 cells (5 × 10^6^ cells resuspended in 100 μL PBS) in each flank. The mice were then treated daily by subcutaneous injection of IL-17C (1 μg/tumor) and/or Ki8751 (10 μg/tumor) near the inoculated tumor site. No animal exhibited signs of toxicity following the administration of IL-17C and/or Ki8751. All inoculations were performed under anesthesia with isoflurane (Hana Pharm, Hwaseong, South Korea) using the Small Animal O_2_ Single Flow Anesthesia System (LMS, Pyeongtaek, South Korea). The concentration of isoflurane was 3% for induction and 2% for maintenance, with 1 L/min oxygen. Inoculations were performed when the mice didn’t respond to physical stimuli when under anesthesia. The size of each tumor was measured daily using digital calipers (Control company, Friendswood, TX, USA) and tumor volume (mm^3^) was calculated as (long diameter)^2^ × (short diameter) × 0.5. On the 10th day, all mice were euthanized using carbon dioxide (CO_2_; with a flow rate 20% per min) and their tumors surgically removed for histological analyses and immunofluorescence (IF) staining. Segments of the excised tumors were immediately fixed in 10% buffered formalin solution (Sigma-Aldrich), embedded in paraffin, and then stained with hematoxylin and eosin (H&E). Histological changes were observed by using a microscope (Olympus Corp., Tokyo, Japan), photographed with Moticam 3.0MP Color Digital Camera (Motic, Causeway Bay, Hong Kong), and analyzed with motic images plus 2.0 software. All animal procedures were approved by the Institutional Animal Care and Use Committee of the Pusan National University (Approval Number: PNU-2016-1380, Approval Date: 5 January 2016).

### 2.13. Enzyme-Linked Immunosorbent Assay (ELISA)

NCM460 and CRC cells (Caco-2, HT-29, DLD-1, and HCT116), and xenograft tumors were stimulated with 100 ng/mL human IL-17C (eBioscience) for 8 h, and the cultured supernatants were collected to detect secreted proteins using the human VEGF ELISA Duo Kits (R&D Systems, Minneapolis, MN, USA). To investigate IL-17C-related cytokines in DLD-1 cells, ELISA was conducted using the human inflammatory cytokines and chemokines multi-analyte ELISArray Kit (Qiagen, MEH-004A), following the manufacturer’s instructions. Sample absorbance values were determined at 450 nm using 540 nm as a reference wavelength, and the standard curve was calculated using a four-parameter logistics curve-fitting algorithm.

### 2.14. IF Staining

Mouse tumor tissues were fixed in 10% buffered formalin solution (Sigma-Aldrich) at room temperature and infiltrated with 20% sucrose overnight at 4 °C. Tumor tissue blocks were then imbedded with OCT Tissue-Tek compound (SAKURA, West Chester, PA, USA) onto a PVC plastic mold and rapidly frozen to −30 °C in dry ice, 15 μm thick sections (at least 10 slices per block) were obtained using a Leica CM1850 cryostat (Leica Microsystems, Wetzlar, Germany). Frozen sections were dried for 2 h at room temperature, washed with PBS three times for 3 min, and surface tensions reduced using an application of 0.05% triton X-100 2 times for 5 min. Sections were blocked in 5% normal goat serum (Jackson ImmunoResearch Laboratories, Inc., West Grove, PA, USA) for 1 h, incubated overnight in a humidified chamber with each primary antibody, 1:200 Rat-anti mouse PECAM (CD31) and Rat-anti mouse VE-Cad (CD144) (Both from BD bioscience, La Jolla, CA, USA), washed with PBS with 0.1% Triton X-100, and then incubated for 1 h with secondary antibodies 1:500 FITC goat anti-rat IgG (BD bioscience) at room temperature. After washing with PBS, samples were mounted with ProLong Gold anti-fade regent containing 4′,6-diamidino-2-phenylindole (DAPI, Invitrogen), and confocal fluorescence images were acquired using the FV10i FLUOVIEW Confocal Microscope (Olympus, Tokyo, Japan).

### 2.15. Immunohistochemistry (IHC)

Formalin-fixed human colon tissues of grade III colorectal adenocarcinoma were obtained (US Biomax, Inc., Derwood, MD, USA). For IHC staining, slides were deparaffinized with xylene (Duksan Pure Chemicals, Kyungki-do, Korea), rehydrated with sequential washes of decreasing concentrations of ethanol (Merck Millipore Corporation, Billerica, MA, USA), and rinsed with tap water (100% xylene 5 min: 2 times, xylene 1:1 with 100% ethanol 5 min: 2 times, 95% ethanol 5 min, 70% ethanol 5 min, 50% ethanol 5 min, tap water). After antigen retrieval and permeabilization, non-specific binding sites were blocked with normal rabbit serum (Vector Laboratories, Burlingame, CA, USA). The slides were then incubated for 2 h in 1:50 diluted IL-17C antibody (Thermo Scientific, Waltham, MA, USA). Antibody binding was detected using a biotinylated secondary antibody and ABC reagent from the Vectastain Elite ABC kit (Vector Laboratories). The slides were developed using the peroxidase substrate solution, counterstained with hematoxylin, and mounted using a VectaMount mounting medium (all from Vector Laboratories). The slides were then observed and photographed with Moticam 3.0 MP Color Digital Camera (Motic).

### 2.16. Western Blot Analysis

Total proteins were boiled at 90 °C for 5 min in Laemmli sample buffer (ELPIS-Biotech, Daejeon, South Korea) at a volume ratio of 4:1. Total protein (20 mg) was separated on 10% sodium dodecyl sulfate-polyacrylamide gel electrophoresis and transferred to polyvinylidene fluoride membranes (Merck Millipore Corporation, Billerica, MA, USA) using a wet transfer system (Hoefer Scientific, Holliston, MA, USA). The membranes were blocked with 5% skimmed milk (BD Bioscience) in Tris-buffered saline 0.05% Tween-20 (20 mM Tris, 140 mM sodium chloride, pH 7.5, and 0.05% Tween 20; all from Amresco, Solon, OH, USA) for 1 h at room temperature. The membranes were then incubated overnight at 4 °C with the primary antibodies, phospho-STAT3 (Tyr705) (1:1000), STAT3 (1:1000), (Cell Signaling Technologies, Danvers, MA, USA), SEMA6D (1:1000) (R&D Systems), and β-actin (1:10000) (Santa Cruz Biotechnology Inc., Santa Cruz, CA, USA). After incubation, the membranes were washed five times for 10–15 min with Tris-buffered saline 0.05% Tween-20 and then incubated with horseradish peroxidase-conjugated anti-rabbit or anti-mouse antibody (1:10000) (both from Enzo Life Sciences, Farmingdale, NY, USA) at room temperature for 1–2 h. Antigen-antibody complexes were visualized using enhanced chemiluminescence reagents (Thermo Scientific).

### 2.17. Statistical Analysis

Statistical analyses were performed using GraphPad Prism version 5.03 for Windows (GraphPad Software, La Jolla, CA, USA). Data are presented as the mean ± SD or the mean ± SEM. The Student’s *t*-test, or one-way or two-way analysis of variance (ANOVA) was used to assess statistical significance; *p* < 0.05 was considered statistically significant.

## 3. Results

### 3.1. IL-17C Enhances Angiogenesis of HIMECs

**Figure 1:**
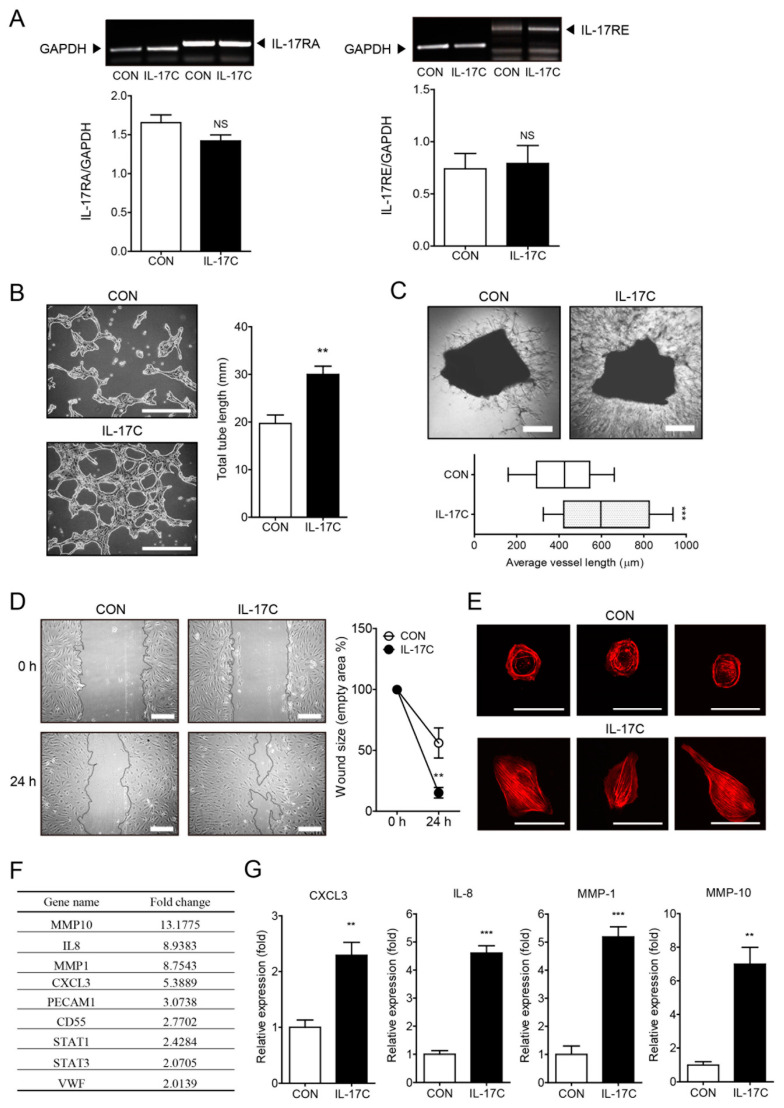
IL-17C stimulation promotes human intestinal microvascular endothelial cells (HIMEC) angiogenesis. (**A**) Representative bands of RT-PCR and quantitative analyses of two receptors of IL-17C, IL-17RA (**A**, **left**), and IL-17RE (**A**, **right**) in HIMECs treated with vehicle control or IL-17C (100 ng/mL, 3 h) are shown (*n* = 3). GAPDH was used for the loading control. NS, not significant. (**B**) HIMECs were seeded into polymerized Matrigel and treated with IL-17C (100 ng/mL, 4 h) for in vitro tube formation assays (**B**, **left**). Scale bar, 100 μm. Total tube length of photos was measured using ImageJ v.1.47 software (*n* = 6) (**B**, **right**). Error bars, SD. ** *p* < 0.01 vs. the control group. (**C**) The mouse aortic ring assay was conducted to investigate ex vivo angiogenic activity of IL-17C (100 ng/mL, 14 d) (**C**, **top**). Scale bar, 50 μm. For quantification, total sprouted tube vessels of 3 independent photos from each group were measured using ImageJ v.1.47 software (**C**, **bottom**). Error bars, SEM (*n* = 3). *** *p* < 0.001 vs. the control group. (**D**) For the wound healing assays, cells were seeded in a culture plate, incubated with culture media with/without IL-17C (100 ng/mL), and scratched to create wounds. Photos were taken 24 h after wound creation (**D**, **left**). Scale bar, 50 μm. A view of representative fields from images of the wound healing assays, analyzed using ImageJ v.1.47 software (**D**, **right**). Error bars, SD (*n* = 6). ** *p* < 0.01 vs. the control group. (**E**) F-actin organization in endothelial cells determined via rhodamine-phalloidin staining (*n* = 3). Scale bar, 50 μm. (**F**) PCR microarray was performed to investigate expression levels of angiogenesis-related genes by IL-17C (100 ng/mL, 3 h) treatment in HIMECs (*n* = 1). (**G**) The mRNA levels of CXCL3, IL-8, MMP-1, and MMP-10 were analyzed by qPCR at 3 h post IL-17C treatment. Error bars, SD (*n* = 3). ** *p* < 0.01 and *** *p* < 0.001 vs. the control group.

IL-17C has been demonstrated to exhibit tumor enhancing activity by increasing epithelial cell survival in CRC [12]. Moreover, IL-17A has been shown to increase tumor angiogenesis and thereby promote tumor progression, including CRC progression [8,10,14]. Therefore, we hypothesized that IL-17C might induce angiogenesis. Having found that HIMECs express the IL-17C receptors IL-17RA and IL-17RE (Figure 1A), we first examined whether IL-17C can induce angiogenesis of HIMECs. Our results showed that IL-17C (100 ng/mL, 4 h) enhanced tube formation of HIMECs cultured on Matrigel (Figure 1B). Similarly, the vessel outgrowth from aortic explants embedded in Matrigel was increased by IL-17C stimulation (100 ng/mL, 14 d) (Figure 1C). Moreover, the results of wound healing assays showed that IL-17C (100 ng/mL, 24 h) promoted cell migration and reduced the overall denuded area compared with the vehicle control (Figure 1D). IL-17C stimulation (100 ng/mL, 15 min) induced F-actin fiber assembly as detected by rhodamine-phalloidin staining indicating increased actin reorganization which is required for cell migration (Figure 1E). Taken together, these results indicate that IL-17C exerts a potent angiogenic activity in vitro and ex vivo, by promoting cell migration.

We next examined the possibility of IL-17C regulating angiogenic factor secretion by HIMECs, which further modulates the angiogenic activity of HIMECs. The microarray results showed that IL-17C (100 ng/mL, 3 h) up-regulated several angiogenic factors, including matrix metalloproteinases (MMP-1 and -10), IL-8, angiogenic CC and CXC chemokines (CCL5 and CXCL3), and endothelial cell markers (PECAM1 and VWF) (Figure 1F). To validate these results, we performed qPCR and found that the mRNA expression levels of CXCL3, IL-8, MMP-1, and MMP-10 were increased by IL-17C (Figure 1G). These results indicate that IL-17C up-regulates the production of an array of angiogenic chemokines, cytokines, and enzymes.

### 3.2. IL-17C Augments the Migratory and Angiogenic Activities of DLD-1

**Figure 2:**
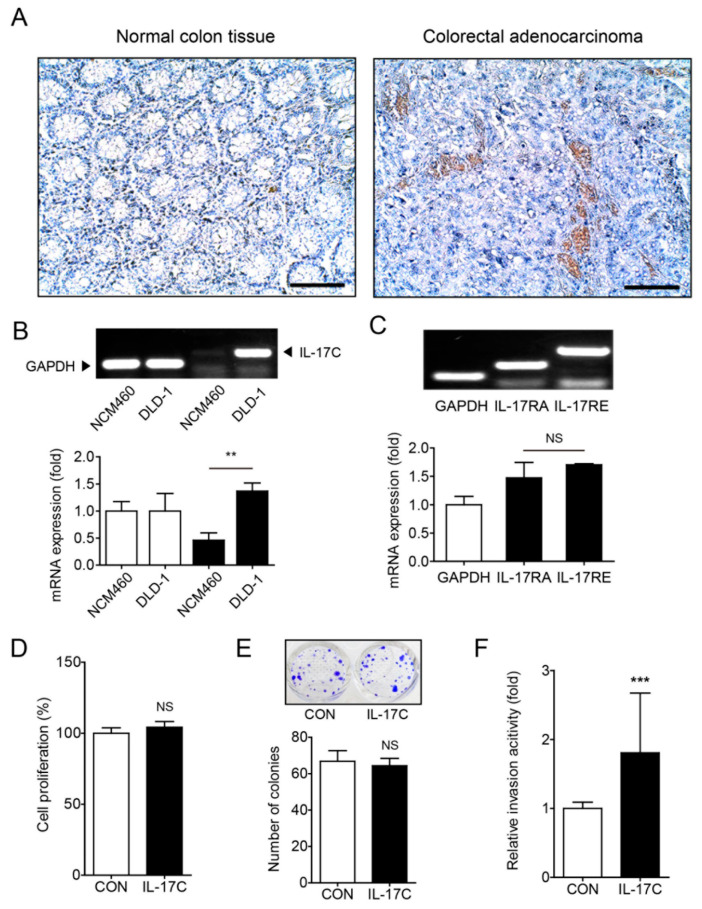

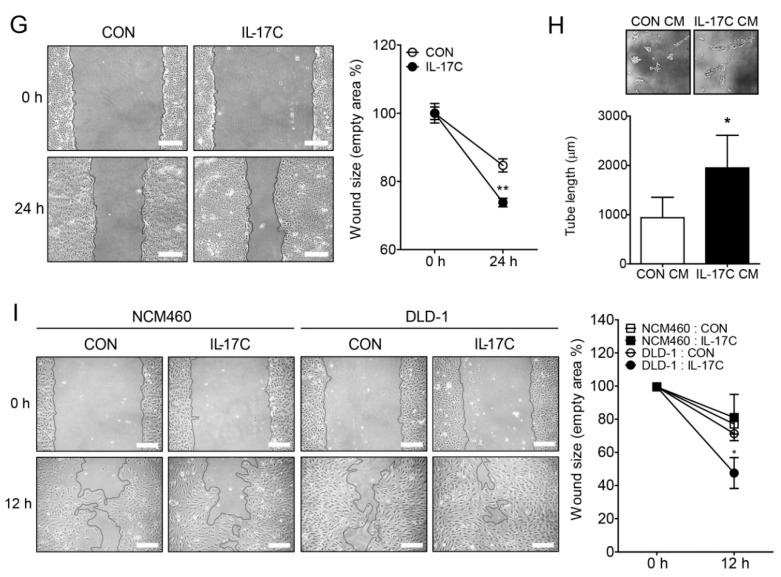
IL-17C has active functions in tumorigenesis. (**A**) Immunohistochemistry staining was used to detect IL-17C expression in human normal colon tissue and colorectal adenocarcinoma (*n* = 3). Scale bar, 100 μm. (**B**,**C**) Representative bands of RT-PCR and quantitative analyses of IL-17C in NCM460 and DLD-1 cells (**B**), and IL-17RA and IL-17RE in DLD-1 cells (**C**) are shown. GAPDH is used as the loading control. Error bars, SD (*n* = 3). ** *p* < 0.01 vs. NCM460 (**B**); NS, not significant (**C**). (**D**,**E**) MTT (**D**) and colony formation (**E**) assays were performed using IL-17C (100 ng/mL, 24 h and 10 d, respectively) stimulated DLD-1 cells. Representative photographs are shown (**E**, top). Error bars, SD (*n* = 6). NS, not significant. (**F**) An invasive assay was performed in DLD-1 cells with IL-17C (100 ng/mL, 24 h) stimulation. Error bars, SD (*n* = 8). *** *p* < 0.001 vs. the control group. (**G**) A migration assay was conducted in DLD-1 cells with IL-17C (100 ng/mL, 24 h) stimulation (**G**, **left**). Scale bar, 50 μm. For quantification, the empty areas of 8 independent photos from each group were measured using ImageJ v.1.47 software (**G**, **right**). Error bars, SD (*n* = 8). ** *p* < 0.01 vs. the control group. (**H**) A tube formation assay was performed with HIMECs by incubating the cells with IL-17C (100 ng/mL, 6 h)-stimulated DLD-1 conditioned medium (CM). Error bars, SD (*n* = 3). * *p* < 0.05 vs. the control CM. The representative bright-field images (magnification, ×200) of tubes are shown (**H**, **top**). Total tube length of photos was measured using ImageJ v.1.47 software (**H**, **bottom**). Error bars, SD (*n* = 3). * *p* < 0.05 vs. the control CM. (**I**) A wound healing assay was conducted in HIMECs incubated with CM from NCM460 or DLD-1 cells with/without IL-17C (100 ng/mL, 12 h) treatment (**I**, **left**). Scale bar, 50 μm. For quantification, the empty areas of 4 independent photos from each group were measured using ImageJ v.1.47 software (**I**, **right**). Error bars, SD (*n* = 4). * *p* < 0.05 vs. the DLD-1 control.

A recent study showed that IL-17C expression increased in human CRC samples and murine tissues of colitis-associated colon cancer, facilitating the development of CRC [12]. In accordance with these results, immunohistochemistry staining showed that IL-17C expression increased in human CRC tissues, compared with normal colon tissues (Figure 2A). Additionally, IL-17C expression increased in DLD-1 cancer cells, compared with NCM460 normal epithelial cells (Figure 2B). DLD-1 cells express the IL-17C receptors IL-17RA and IL-17RE (Figure 2C). Although the results of the previous study indicated that IL-17C increases IEC survival, the results of MTT (24 h) and colony forming assays (10 d) showed that IL-17C (100 ng/mL) failed to increase cell proliferation in DLD-1 cells (Figure 2D,E). However, IL-17C (100 ng/mL) facilitated cell migration in invasion (48 h) and wound healing assays (24 h) (Figure 2F,G). These results indicate that IL-17C increases cancer cell invasiveness and migration ability in vitro, rather than promoting cell proliferation.

IL-17C increased angiogenesis of intestinal endothelial cells, acting as a novel angiogenic factor (Figure 1). Given that cell-cell interaction between cancer and endothelial cells is crucial for tumor angiogenesis, we investigated whether cancer cell-derived factors could affect angiogenesis in endothelial cells and thus IL-17C stimulation modulates the secretion of those factors by DLD-1 cells. To address this, conditioned medium (CM) from IL-17C- or vehicle (CON)-treated DLD-1 cells was collected to treat HIMECs. IL-17C CM increased tube formation and HIMEC migration, compared with CON CM, suggesting that IL-17C stimulated the secretion of some angiogenic factors by DLD-1 cells (Figure 2H,I, respectively).

### 3.3. IL-17C Increases VEGF Production in DLD-1 Cells

**Figure 3:**
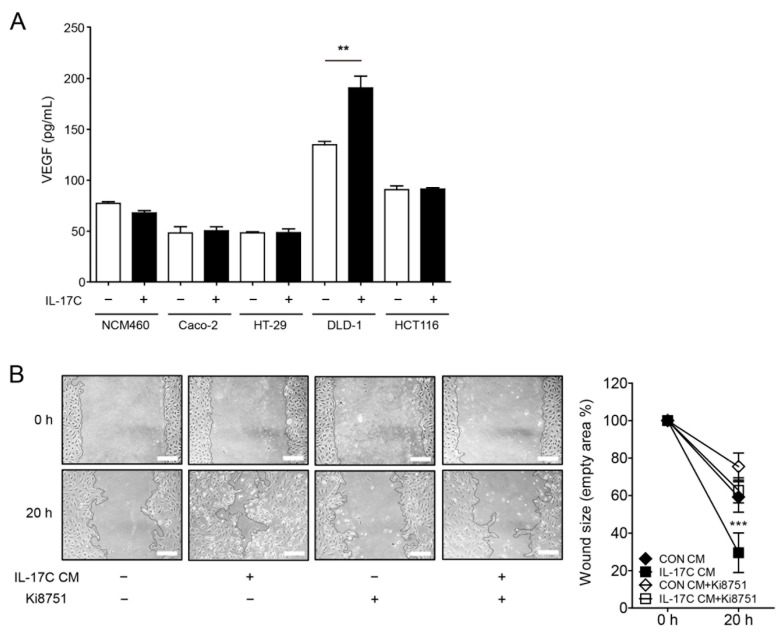
IL-17C-induced cell migration is mediated by VEGF and suppressed by the VEGFR2 inhibitor Ki8751. (**A**) Human VEGF concentration was calculated in IL-17C (100 ng/mL, 8 h)-treated CM from NCM460, Caco-2, HT-29, DLD-1, and HCT116 cells by using ELISA. Error bars, SD (*n* = 3). ** *p* < 0.01 vs. the DLD-1 control group. (**B**) HIMECs were pretreated of Ki8751 (4.7 μg/mL) for 30 min, scratched, and incubated in CM from IL-17C (100 ng/mL)-stimulated DLD-1 cells for 20 h (**B**, **left**). Scale bar, 50 μm. Representative fields from photos of the wound healing assays, analyzed using ImageJ v.1.47 software (**B**, **right**). Error bars, SD (*n* = 6). *** *p* < 0.001 vs. the control CM group.

We examined the possibility that IL-17C modulated the production of angiogenic VEGF by DLD-1 cells. Interestingly, IL-17C selectively up-regulated the production of DLD-1-derived VEGF, with no change in VEGF production in normal colonic epithelial NCM460 cells nor other CRC cell lines, including Caco-2, HT-29, and HCT116 cells (Figure 3A). Moreover, IL-17C failed to modulate the production of other angiogenic cytokines, including IL-1α, IL-1β, IL-6, and IL-8, and anti-angiogenic factors, including IFN-γ and TNF-α, in DLD-1 cells (Supplementary Figure S1A,B, respectively). To confirm these results, we demonstrated that pretreatment with Ki8751, a selective inhibitor of VEGFR2, inhibited IL-17C CM-induced HIMEC migration (Figure 3B).

### 3.4. IL-17C Promotes the In Vivo Growth of DLD-1 Cells Via VEGF Production/Angiogenesis

**Figure 4:**
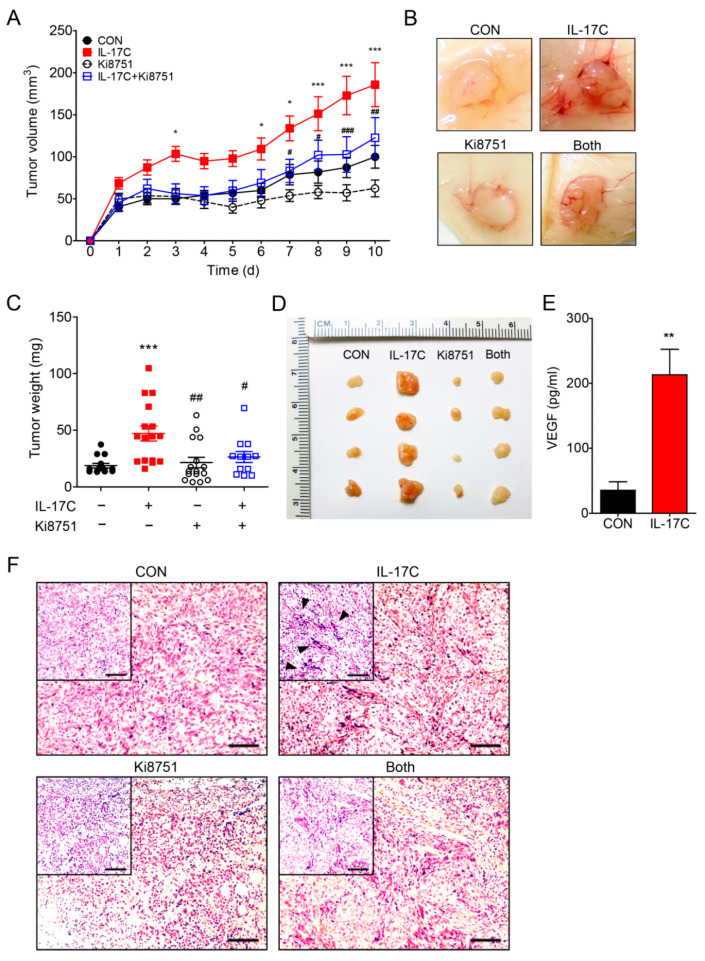

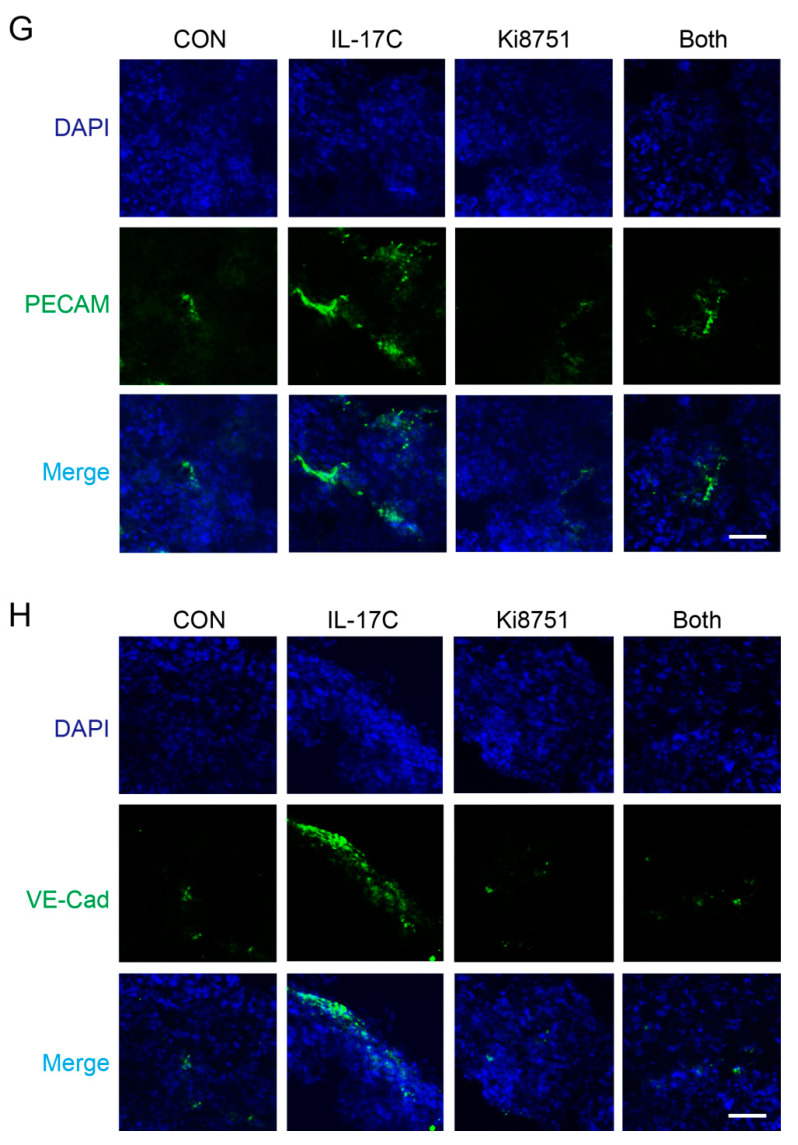
VEGFR2 inhibition suppresses IL-17C-induced tumor progression and angiogenesis in vivo. (**A**–**D**) A mouse xenograft experiment was conducted with treatment with IL-17C (1 μg/tumor) and/or Ki8751 (10 μg/tumor) by subcutaneous injection (CON, *n* = 10; IL-17C, *n* = 10; Ki8751, *n* = 10; Both, *n* = 9). Tumor volume was analyzed daily by using a caliper, and after euthanasia (**A**), and the representative photographs of the gross tumors in mice are shown (**B**). Tumor weight was also measured (**C**), and the photographs of the xenografts are shown (**D**). * *p* < 0.05 and *** *p* < 0.001 vs. the control group. # *p* < 0.05, ## *p* < 0.01, and ### *p* < 0.001 vs. the IL-17C group. Error bars, SEM. (**E**) The protein levels of human VEGF in tumor tissues were analyzed using ELISA. ** *p* < 0.01 vs. the control group. Error bars, SEM. (**F**) Tumor tissue sections from each group were stained with H&E. Scale bar, 100 μm. (**G**,**H**) Tumor tissue sections from each group were stained for FITC-labeled PECAM (**G**) and FITC-labeled VE-cad (**H**). DAPI staining was applied to identify nucleus. Scale bar, 50 μm.

In order to address whether IL-17C confers tumor-promoting activities in the in vivo model of colorectal cancer, DLD-1 cells were subcutaneously inoculated into the flanks of nude mice (*n* = 10 per group) and IL-17C (1 μg/tumor) was injected peritumorally for 10 days. IL-17C-treated tumors grew bigger and faster, compared with than vehicle-treated tumors, resulting in substantially increased tumor volumes (Figure 4A). The representative photographs of the gross tumors in mice are shown in Figure 4B. Furthermore, weights and sizes of IL-17C-treated tumors were markedly increased, compared with the control tumors (Figure 4C,D). Given that IL-17C selectively increased the secretion of angiogenic VEGF by DLD-1 cells, we postulated that increased growth of IL-17C-treated DLD-1 cells was mediated by enhanced angiogenesis. Moreover, VEGF expression increased in IL-17C-treated tumors, compared with the vehicle-treated tumors (Figure 4E). To delineate the role of upregulated VEGF production in tumorigenesis of IL-17C-treated xenografts, we used the VEGFR2 inhibitor Ki8751 (10 μg/tumor, peritumoral injection) to block the biological activity of VEGF. As illustrated in Figure 4A–D tumors co-treated with Ki8751 and IL-17C grew more slowly than those treated with IL-17C alone. Thus, in monitoring tumor growth in the presence of Ki8751, we found that co-treatment with Ki8751 suppressed IL-17C-induced tumor growth. Moreover, histological analysis of IL-17C-treated xenografts showed increased tumor vascularity, while Ki8751 co-treatment suppressed the angiogenic effect of IL-17C (Figure 4F).

During IF staining, the expression of the endothelial cell adhesion molecules platelet endothelial cell adhesion molecule (PECAM)-1 and vascular endothelial cadherin (VE-cad) was significantly increased in the tumors of the IL-17C-injected group, but ameliorated by Ki8751 co-stimulation (Figure 4G,H). These results indicated that IL-17C promotes tumor progression by upregulating VEGF production, which increases endothelial cell activation and tumor angiogenesis, both in vitro and in vivo.

### 3.5. IL-17C Upregulates miR-23a-3p Which Enhances VEGF Production and Suppresses SEMA6D Target Gene Expression

**Figure 5:**
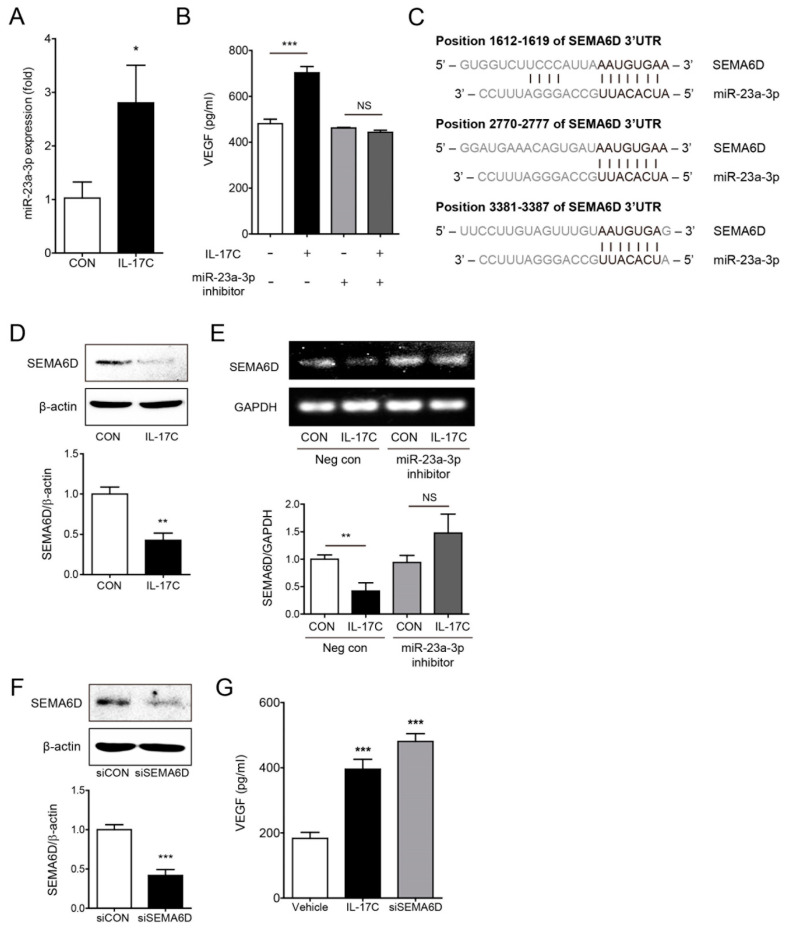
IL-17C-induced miR-23a-3p expression mediates VEGF secretion by targeting SEMA6D. (**A**) The expression of miR-23a-3p is higher in IL-17C (100 ng/mL, 3 h)-treated DLD-1 cells. Error bars, SD (*n* = 3). * *p* < 0.05 vs. the control group. (**B**) ELISA was used to determine the level of IL-17C-induced VEGF concentration in miR-23a-3p inhibitor-transfected DLD-1 cells. Error bars, SD (*n* = 4). *** *p* < 0.001; NS, not significant. (**C**) Target gene prediction was analyzed by two independent miRNA target databases. There are sequences of three predicted binding positions (black letter) of miR-23a-3p within 3′-UTR of SEMA6D mRNA. (**D**) Representative bands of Western blotting and quantitative analyses of SEMA6D protein expression in DLD-1 cells after IL-17C (100 ng/mL, 8 h) stimulation are shown. β-actin was used as a loading control. Error bars, SD (*n* = 3). ** *p* < 0.01 vs. the control group. (**E**) RT-PCR was conducted for comparing the alteration of SEMA6D expression in miR-23a-3p inhibitor-transfected DLD-1 cells stimulated with IL-17C to that in the negative control DLD-1 cells. GAPDH was used as a loading control. The bar graph below shows the result of quantitative analyses. Error bars, SD (*n* = 3). ** *p* < 0.01; NS, not significant. (**F**) Representative bands of Western blotting and quantitative analyses of SEMA6D protein expression after siSEMA6D transfection are shown. Error bars, SD (*n* = 3). *** *p* < 0.001 vs. the siCON group. (**G**) Secreted human VEGF levels in siSEMA6D-transfected DLD-1 cells were analyzed using ELISA. Error bars, SD (*n* = 4). *** *p* < 0.001 vs. the control group.

Growing evidence suggests that miRNAs play important roles in angiogenesis [17]. To determine the specific molecular mechanism through which IL-17C activates VEGF-induced tumor angiogenesis, the expression of well-known angiogenic miRNAs was analyzed in IL-17C-treated DLD-1 cells. Among them, miR-23a-3p expression was considerably increased in DLD-1 cells by IL-17C treatment (Figure 5A). As expected, miR-23a-3p inhibition by transfection of its inhibitor suppressed IL-17C-induced VEGF induction in DLD-1 cells (Figure 5B). Furthermore, to identify the genetic target of miR-23a-3p, we predicted target candidates using DIANA-microT v3.0 and TargetScan algorithms programs, and miRNA binding efficacies to the 3′UTR regions of the target genes were investigated. Based on our results, SEMA6D is the strongest miR-23a-3p genetic target of all candidate genes. The three positions of SEMA6D 3′UTR highly paired with the 7 or 8 mer sequence of miR-23a-3p UUACACUA (Figure 5C). IL-17C treatment strongly suppressed the expression of SEMA6D in DLD-1 cells, supporting that decreasing SEMA6D is closely related to downstream of the IL-17C signaling (Figure 5D). IL-17C-induced SEMA6D suppression was recovered by inhibition of miR-23a-3p expression in DLD-1 cells (Figure 5E). Similarly, inhibition of SEMA6D expression by siRNA transfection increased VEGF induction in DLD-1 cells (Figure 5F). These results indicate that miR-23a-3p activation by IL-17C treatment is necessary for inducing VEGF production by SEMA6D target gene suppression.

### 3.6. STAT3 Activation is Required for IL-17C-Induced miR-23a-3p Expression

**Figure 6:**
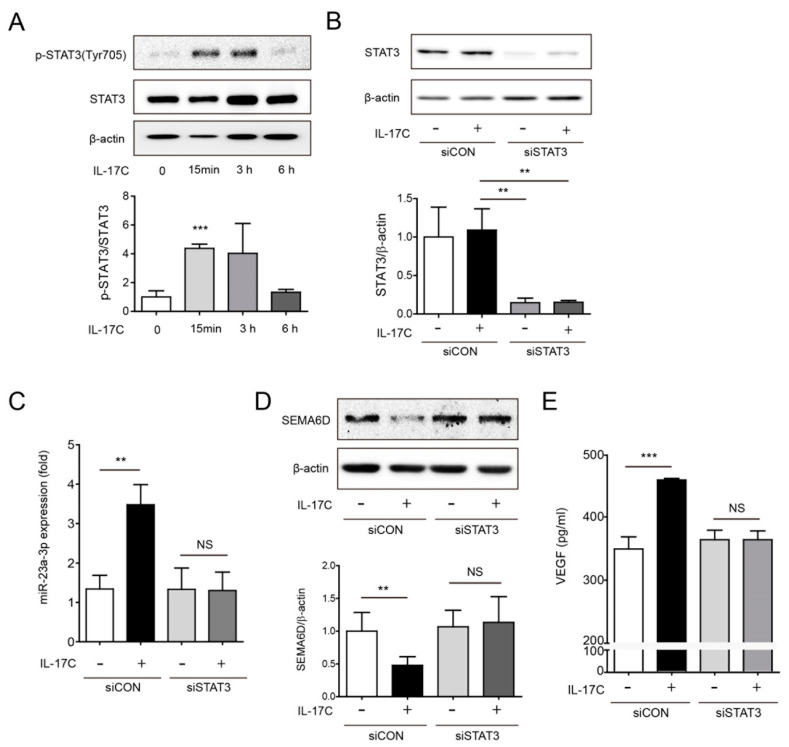
IL-17C activates STAT3 signaling. (**A**) Representative bands of Western blotting and quantitative analyses of STAT3 phosphorylation by IL-17C stimulation in DLD-1 cells are shown. STAT3 and β-actin were used as a loading control. Error bars, SD (*n* = 3). *** *p* < 0.001 vs. the 0 h group. (**B**) STAT3 protein expression was shown in cells transiently transfected with siRNA of STAT3. The bar graph below shows the result of quantitative analyses. Error bars, SD (*n* = 3). ** *p* < 0.01. (**C**) miR-23a-3p expression in transiently transfected DLD-1 cells with siSTAT3 was measured using qPCR. Error bars, SD (*n* = 3). ** *p* ≤ 0.01; NS, not significant. (**D**) SEMA6D protein expression in siSTAT3-transfected and IL-17C-stimulated DLD-1 cells was detected using Western blotting. The results of quantitative analyses are shown below. β-actin was used as a loading control. Error bars, SD (*n* = 3). ** *p* < 0.01. (**E**) ELISA was conducted to determine VEGF levels in siSTAT3-transfected DLD-1 cells. Error bars, SD (*n* = 4). *** *p* < 0.001; NS, not significant.

Previous reports showed that IL-17A-induced STAT3 activation promotes tumor progression and angiogenesis in hepatocellular carcinoma and non-small-cell lung cancer, respectively [10,23]. Moreover, another study reported that STAT3-mediated miR-23a expression leads to decreased gluconeogenesis in hepatocellular carcinoma [24]. Therefore, we investigated whether STAT3 activation is required for IL-17C-mediated miR-23a-3p expression in DLD-1 cells. STAT3 phosphorylation was induced by IL-17C stimulation at tyrosine residue (Tyr705), approximately 15 min after stimulation, and STAT3 remained phosphorylated up to 3 h (Figure 6A). To test if STAT3 phosphorylation regulates miR-23a-3p expression, STAT3 siRNA (siSTAT3) experiments were performed. Transfection of DLD-1 cells with siSTAT3 inhibited STAT3 expression (Figure 6B). Intriguingly, upregulation of miR-23a-3p by IL-17C treatment was not observed by siSTAT3 transfection, suggesting that STAT3 activation is necessary for IL-17C-mediated miR-23a-3p induction (Figure 6C). Moreover, SEMA6D suppression is also related to STAT3 activation, resulting that IL-17C did not inhibit SEMA6D in STAT3 knockdown experiments (Figure 6D). Furthermore, IL-17C-induced VEGF expression was significantly inhibited by siSTAT3 transfection (Figure 6E). These results suggest that IL-17C-mediated miR-23a-3p expression may require STAT3 activation which subsequently regulates SEMA6D and VEGF expression.

## 4. Discussion

**Figure 7:**
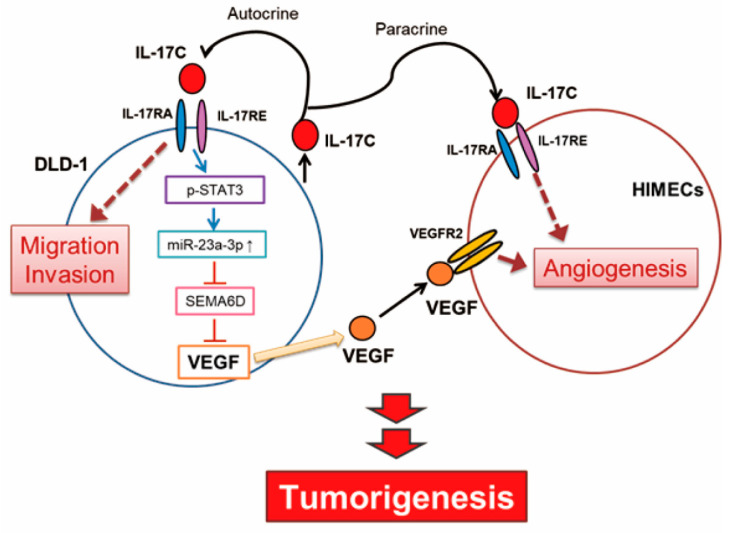
A schematic shows IL-17C-mediated molecular network and how miR-23a-3p regulates IL-17C-induced tumor angiogenesis in colorectal cancer by promoting VEGF production.

The IL-17C family may also act as a double-edged sword by playing a dual role as both oncogene and tumor suppressor in tumor development as suggested by Qian et al. [5]. Infection and acute inflammation in the gut can induce IL-17C upregulation to maintain homeostasis and mediate beneficial pro-inflammatory responses [7,25]. This suggests that IL-17C is required in normal gastrointestinal function, because pro-inflammatory effect and host defense response protects the body from harmful injury. In contrast, chronic inflammation can make the intestinal environment more susceptible to disease. In such an environment, IL-17C can be conducive to tumorigenesis, similar to other pro-inflammatory cytokines. In the present study, IL-17C can promote angiogenesis and migration in malignant colorectal cancer cell, DLD-1, suggesting that IL-17C contributes to the steps of tumorigenesis in chronic tumor environment (Figure 7).

Our data indicated that IL-17C promoted tumor angiogenesis in CRC. IL-17C showed potent angiogenic activity in several functional experiments such as migration assay, F-actin staining, in vitro tube formation assay, and ex vivo mouse aortic ring assay (Figure 1). Additionally, several angiogenic factors such as MMPs and chemokines were significantly increased by IL-17C stimulation in HIMECs (Figure 1). Moreover, the angiogenic activity of IL-17C was found to be highly associated with colorectal cancer cell malignancy in vitro and increased tumorigenesis in vivo (Figure 2 and Figure 4). IL-17C further increased DLD-1 cell malignancy by increasing their invasiveness and migration activity, rather than promoting cancer cell proliferation and survival (Figure 2). These results are consistent with those of previous studies, showing that the IL-17 family can induce tumor angiogenesis without significantly affecting cell proliferation [10,14,26]. These results indicate that angiogenesis is the key mechanism of IL-17C’s modulation of CRC progression.

The intestinal epithelial cell-derived angiogenic factors by IL-17C stimulation can cause intestinal endothelial cells to promote angiogenesis in tumor environment. We therefore characterized the putative angiogenic factors secreted from IL-17C-stimulated CRC cell lines. VEGF is a chief angiogenic factor, essential to the regulation of tumor angiogenesis and vascular permeability [27]. IL-17C stimulation induced VEGF expression in CRC. Specifically, IL-17C-induced VEGF induction was observed in the DLD-1 cells, but not in the other CRC cells and normal intestinal epithelial cells (Figure 3). Meanwhile, the basal level of VEGF in DLD-1 was higher than the levels of other cell lines without any stimulation. It is well known that DLD-1 is one of the most malignant cancer cell lines from male colorectal adenocarcinoma with microsatellite instability and mutations in KRAS, PI3KCA, and TP53 [28,29]. In this regard, increased VEGF levels in DLD-1 cells than other CRC cells may be partly contributed by their high malignancy. ELISA was equally conducted to screen for angiogenesis-related cytokines. Among them, IL-8, IL-6, IL-1α, and IL-1β are well known pro-angiogenic factors [30,31,32]. IFN-γ and TNF-α have been shown to contribute to anti-tumor activity by exerting anti-angiogenic effects [33]. Having found that no changes were observed in these angiogenesis-related factors after IL-17C stimulation in DLD-1 cells (Supplementary Figure S1), the angiogenic effect of IL-17C on DLD-1 cells can be mostly mediated by VEGF signaling. Furthermore, the levels of secreted human VEGF were increased by IL-17C injection in tumor tissues (Figure 4), indicating that IL-17C can also promote tumor progression in in vivo mouse xenograft models through IL-17C-stimulated human VEGF production in DLD-1 cells. Because human VEGF can induce angiogenesis in other species, IL-17C-induced increase of human VEGF effectively promoted tumor angiogenesis by using and recruiting mouse endothelial cells without mouse VEGF expression in the mouse xenograft model [34]. Having found the critical role of VEGF in IL-17C-induced angiogenesis, we sought for the possibility that IL-17C-induced VEGF of epithelial origin can affect endothelial cells in the tumor microenvironment. As expected, CM of IL-17C-stimulated DLD-1 cells promoted endothelial cell migration and tube formation (Figure 2). Given that this increased migration activity was suppressed by the VEGFR2 inhibitor Ki8751 pretreatment (Figure 3), we are convinced that VEGF is the key molecule in epithelial and endothelial cell communication during IL-17C signaling in tumor angiogenesis.

IL-17C-induced VEGF production was found to be closely related to STAT3 signaling pathway that promotes tumor growth [10,23,35,36]. STAT3 signaling is considered an important molecular mechanism of the IL-17 family-associated tumorigenesis [10,23]. Phosphorylation of STAT3 in serine and tyrosine residues was dependent on Janus kinase and MAPK activation, respectively [37]. We found that IL-17C stimulation significantly increased STAT3 phosphorylation, and transfection of siSTAT3 suppressed IL-17C-induced VEGF production (Figure 6), providing evidence that STAT3 signaling is the initial action of IL-17C signaling in CRC.

In the present work, we showed a specific molecular mechanism by which IL-17C regulates tumor angiogenesis in CRC. Several methods are currently used for detecting individual miRNAs, and microarrays are the most common strategies for miRNA profiling [38]. The miR-23~27~24 clusters are already known as “AngiomiRs,” as they enhance angiogenesis and expressed in microvascular endothelial cells [17]. Moreover, the miR-23~27~24 clusters were shown to promote angiogenesis by targeting Sprouty2 and SEMA6A proteins, which exert antiangiogenic activity [21]. SEMA6D is a semaphorin related to immune responses, heart development, organogenesis, developmental angiogenesis, and cancer [39,40]. SEMA6D expression is associated with enhanced breast invasive carcinoma patient survival, suggesting that SEMA6D is closely associated with tumor angiogenesis [41]. Specifically, silencing SEMA6D enhanced pericyte adhesion and barrier function of an endothelial-pericyte co-culture system [42]. Moreover, there is obvious evidence that semaphorin signaling reduced angiogenic potential by antagonizing the proangiogenic activity of VEGF [43,44]. We found that increased IL-17C-induced miR-23a-3p expression can suppress anti-angiogenic activity of SEMA6D and increase VEGF induction to promote tumor growth and tumor angiogenesis (Figure 5). Moreover, STAT3 phosphorylation is associated with IL-17C activation and transfection of siSTAT3 did not promote miR-23a-3p induction (Figure 6). Inhibition of STAT3 restored IL-17C-induced SEMA6D expression and decreased IL-17C-induced VEGF induction (Figure 6), further reaffirming that SEMA6D downregulation by miR-23a-3p is the downstream molecular mechanism of STAT3 phosphorylation in IL-17C signaling.

In conclusion, we have identified that IL-17C promotes tumor angiogenesis in CRC by activating intestinal epithelial and endothelial cells in an autocrine and paracrine manner. The mechanisms for angiogenic activity of IL-17C involve activation of the molecular cascade of the STAT3/miR-23a-3p/SEMA6D axis and subsequent production of VEGF. The present findings provide important information of that IL-17C can be classified as a new angiogenic factor, which could lead to new avenues for the treatment of CRC.

## Supplementary Materials

The following are available online at https://www.mdpi.com/2073-4409/9/6/1363/s1, Figure S1: Secretion of other angiogenic-associated cytokines is not significantly changed by IL-17C, Figure S2: Representative bands and two other repeats of RT-PCR of Figure 1A are shown, Figure S3: IHC images of Figure 2A and representative bands and two other repeats of RT-PCR of Figure 2B and C are shown, Figure S4: Representative bands and two other repeats of Western blotting of Figure 5D and representative bands and two other repeats of RT-PCR of Figure 5E are shown, Figure S5: Representative bands and two other repeats of Western blotting of Figure 6A, B, and D are shown.
