# Supplementary material for: miR-23a-3p is a Key Regulator of IL-17C-Induced Tumor Angiogenesis in Colorectal Cancer
Source: Cells. 2020 Jun 1;9(6):1363. doi: 10.3390/cells9061363 (PMC7348989; doi:10.3390/cells9061363)
Supplement: Supplementary file 1 [file cells-09-01363-s001.pdf]

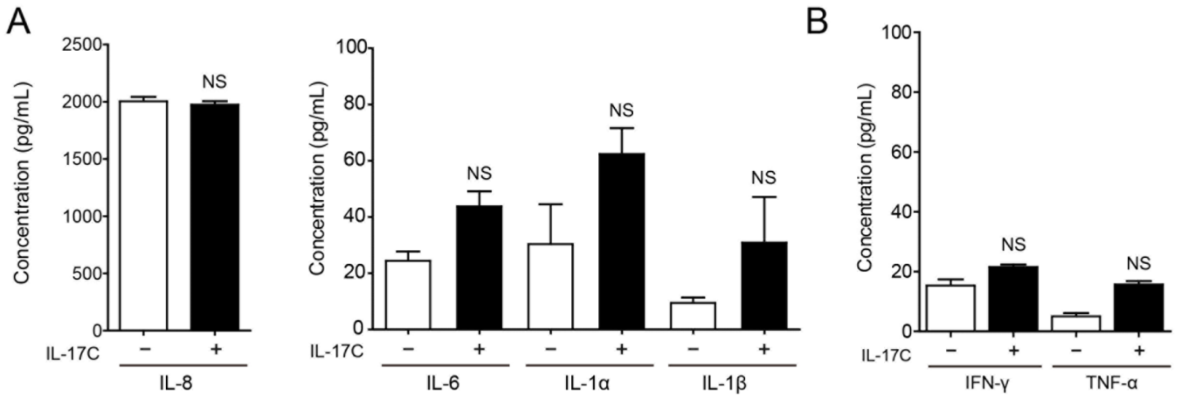

Supplementary figure S1. Secretion of other angiogenic-associated cytokines is not significantly changed by IL-17C. (A and B) ELISA protein array was performed to identify and quantify the levels of the secreted proteins such as IL-8, IL-6, IL-1α, IL-1β, IFN-γ, and TNF-α in DLD-1 cells treated with IL-17C (100 ng/ml) for 8 h. Error bars, SD (n=3), NS, not significant.

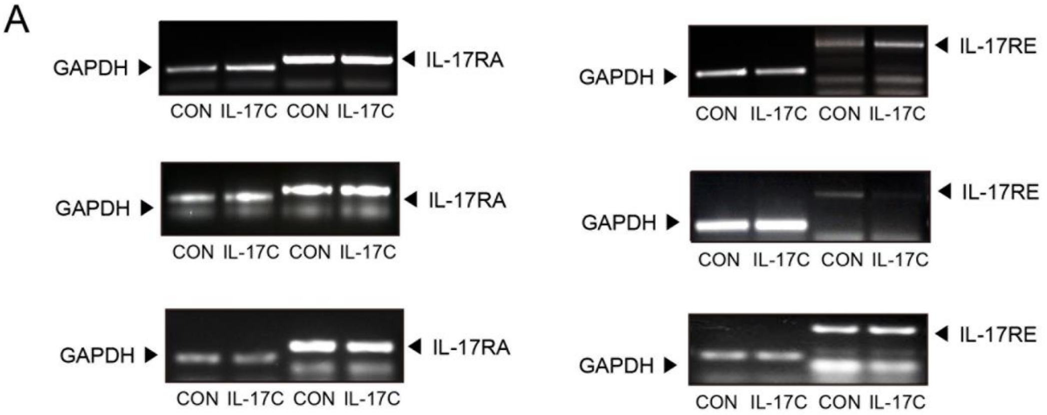

Supplementary figure S2. Representative bands and two other repeats of RT-PCR of Figure 1A are shown.

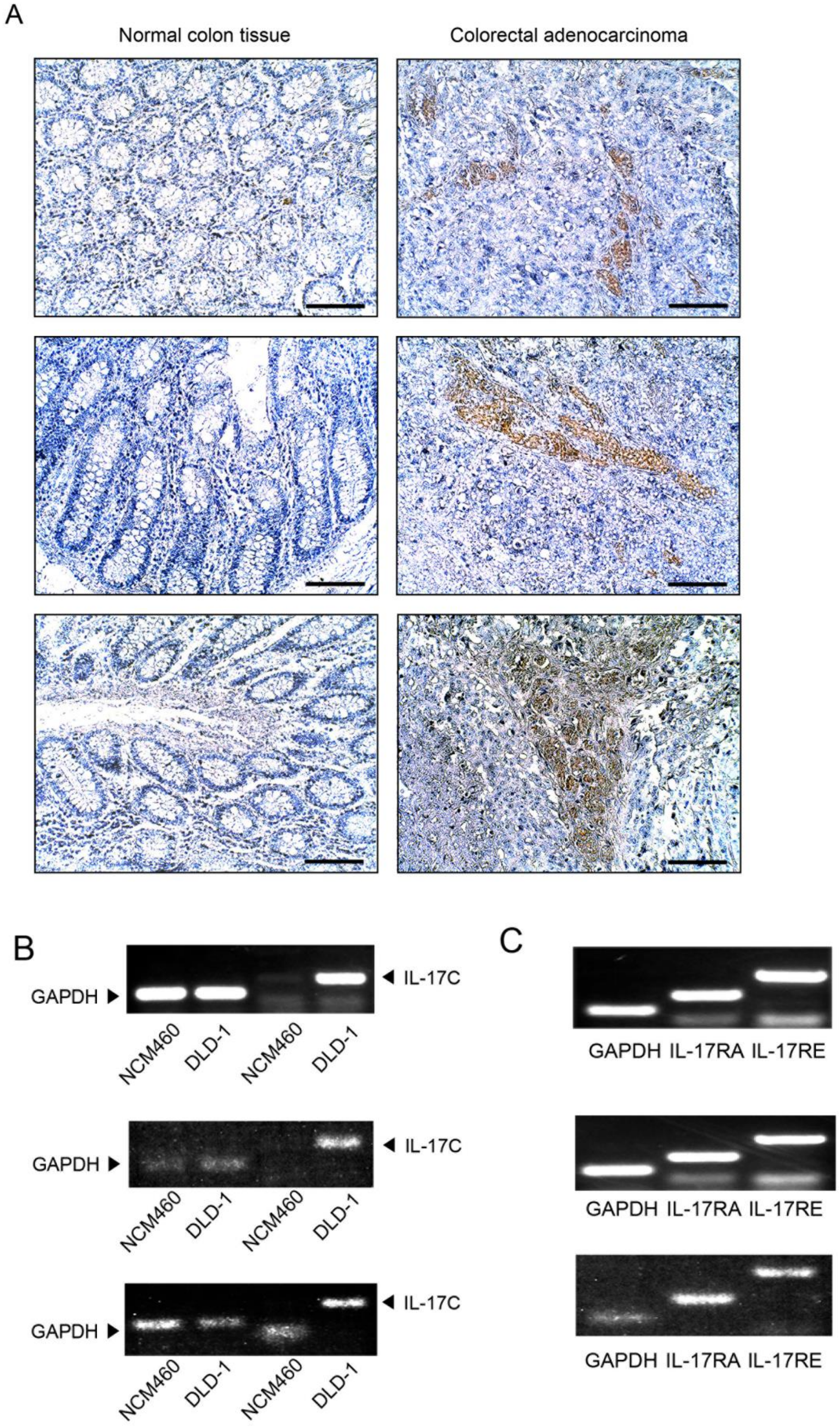

Supplementary figure S3. IHC images of Figure 2A and representative bands and two other repeats of RT-PCR of Figure 2B and C are shown.

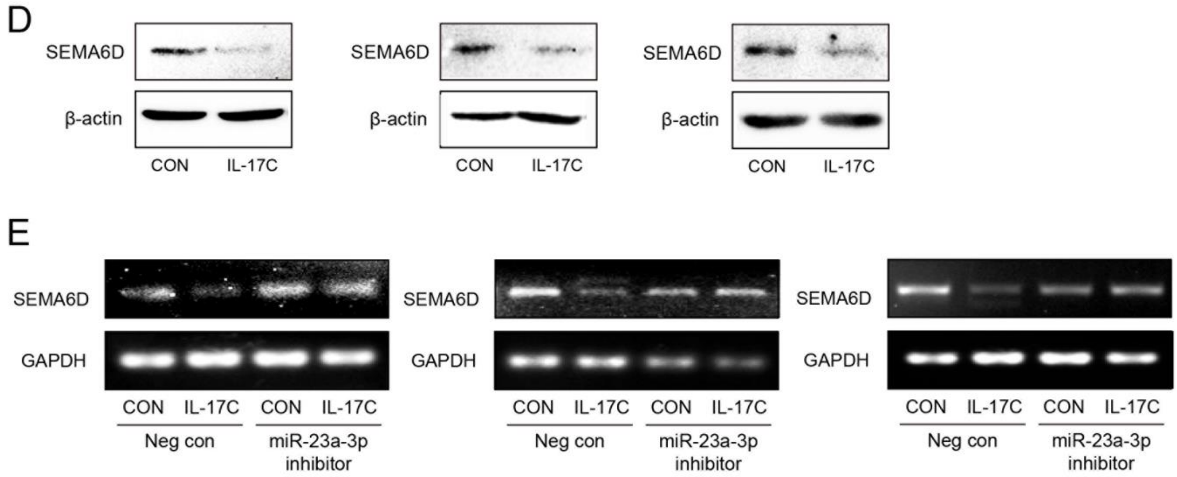

Supplementary figure S4. Representative bands and two other repeats of Western blotting of Figure 5D and representative bands and two other repeats of RT-PCR of Figure 5E are shown.

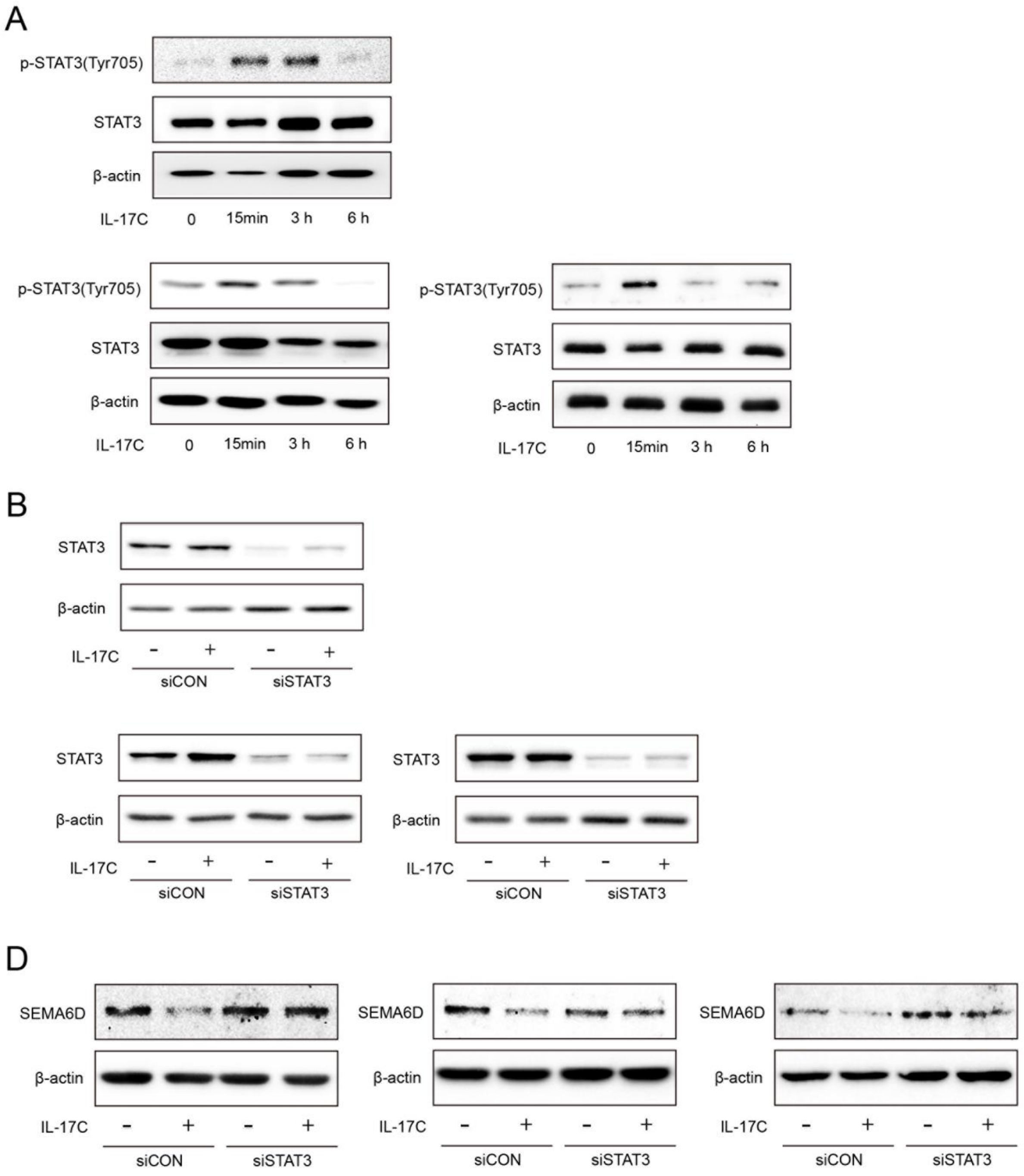

Supplementary figure S5. Representative bands and two other repeats of Western blotting of Figure 6A, B, and D are shown.
